# Internal evaluation of medical programs is more than housework: A scoping review

**DOI:** 10.1371/journal.pone.0305996

**Published:** 2024-10-25

**Authors:** Sujani Kodagoda Gamage, Tanisha Jowsey, Jo Bishop, Melanie Forbes, Lucy-Jane Grant, Patricia Green, Helen Houghton, Matthew Links, Mark Morgan, Joan Roehl, Jessica Stokes-Parish

**Affiliations:** Faculty of Health Sciences and Medicine, Bond University, Robina, QLD, Australia; Bilawal Medical College, Liaquat University of Medical and Health Sciences, PAKISTAN

## Abstract

**Purpose:**

The aim of this scoping review was to explore current program evaluation practices across various medical schools.

**Methods:**

We conducted searches in MEDLINE (Ovid), Embase (Elsevier) and ERIC (ed.gov) for original research and review articles related to medical education evaluation with key words *evaluation*, *program*, *medical education*, *pre-registration*, *framework*, *curriculum*, *outcomes*, *evaluation*, *quality*. We followed Arksey and O’Malley’s (2005) process for scoping reviews.

**Results:**

Thirty-two articles were included. Studies were primarily concerned with either proving (n = 21) or improving efficacy of their programs (n = 11). No studies aimed at comparing programs. Nine were literature reviews. Others aimed to develop a new evaluation model (n = 7) or apply (n = 12) or validate (n = 4) an existing model (or part thereof). Twenty-two studies explicitly identified an evaluation model they had used or would recommend. Most frequently used models for evaluation were: Context-Input-Process-Product, Kirkpatrick, World Federation Medical Education, and the Standards by Joint Committee on Standards for Educational Evaluation. Overall, evaluations were learner-focused and accreditation driven with a minority considering the broader influences of program success.

**Conclusion:**

Program evaluation is fundamental to driving the quality of education delivered to produce workforce**-**ready healthcare professionals. The focus of current evaluations is on student experience and content delivery with a significant gap in the existing literature on evaluation related to staff, learner/staff well-being, equity, diversity, and meta evaluation.

## Introduction

Housework is an unsung hero behind a flawless home. Similarly, internal program evaluation is an unrecognized champion behind successful medical schools. Evaluation is considered foundational to ensuring program quality, yet it is often overlooked for major awards or grants. Alarmingly, like housework, program evaluation is considered a tedious task. Getting program evaluation wrong risks reputational decay and accreditation loss. The problems of this paradox are many, not least of which is that newcomers to program evaluation often find themselves navigating unfamiliar territory without clear guidance on evaluation methodologies or tools However, considering the critical importance of medical programs for ensuring the preparedness of future clinicians, undertaking effective evaluation with appropriate evaluation models and methodologies is paramount.

Program evaluation is the “systematic collection and analysis of information related to the design, implementation, and outcomes of a program, for the purpose of monitoring and improving the quality and effectiveness of the program” [1]. That is, program evaluation is concerned with unveiling the factors that shape the success of the program and the actions needed to increase success [2]. In this study, we focus on internal evaluation of pre-registration medical programs where the associated degree leads to registration eligibility as a medical doctor by the respective medical body. The term pre-registration can be applied to both undergraduate and postgraduate medical programs.

Considering the housework metaphor, we invite readers to think about models for program evaluation as cleaning products and areas to be evaluated as areas to be cleaned. Quality products (models) will not do the housework, but will make the process easier. Like in housework, there is no universal product (model) or process that suits all programs. Hence, people undertaking program evaluation often reach for a widely acknowledged model to guide them, such as the Context-Input-Process-Product (CIPP) [3], which is a versatile cyclical evaluation framework that offers the flexibility to be adjusted as needed, enabling the identification of errors or deficiencies at each stage. It informs decision-making about program planning, organization, implementation, and enhancement. Another example is the Kirkpatrick Model, which is used to evaluate predetermined outcomes via attitudes, knowledge and skills, behaviour change and overall program results according to four levels of learning [4, 5]. Its modified version, The New World Kirkpatrick model, incorporates in-depth outcome analysis components at each level [6]. These are just three models among many.

Despite the substantive literature available, there remains a lack of consensus regarding approaches to medical school program evaluation. In the context of this broad topic, a scoping review is more appropriate than a systematic review to explore the literature to map the key concepts and to identify gaps in literature. We undertook this scoping review to explore why, how and, what medical schools evaluate within their pre-registration medical programs.

### Research questions

1. *Why* were pre-registration medical programs evaluated?
2. *How* do pre-registration medical programs get evaluated?
3. *What* aspects of pre-registration medical programs get evaluated?

## Methods

Given the diversity of program evaluation approaches and ways in which they are reported, we decided that a scoping review was the best approach to literature review. Our scoping and analytical approaches were based on constructivism and guided by Arksey and O’Malley [7]. The five essential stages suggested for an effective scoping review are: identify research question, identify relevant studies, study selection, chart data and collate, summarise, and report results.

## Search strategy and identification of relevant studies

A topic specialist librarian guided our search strategy. The search string is available in S1 File. The final search was conducted in May 2024. We combined keywords and subject heading terms for MEDLINE (Ovid), and then adapted for Embase (Elsevier) and ERIC (ed.gov) using the Polyglot Search Translator [8]. Inclusion and exclusion criteria are outlined in Table 1. In addition, we screened reference lists of literature that met our inclusion criteria.

**Table 1:** Inclusion and exclusion criteria.

Inclusion Criteria	Exclusion Criteria
• Research article/discussion article/ methodology article. • Texts focused on evaluation of pre-registration medical program or part thereof (e.g. program domain). • Episodic program evaluation. • Texts describing a model/steps/guidelines used to evaluate a program. • Evaluation which includes stakeholder perspective (e.g. student, academic staff). • Research published from 2000–2024	• Conference abstract, poster or presentation, opinion piece. • Full text not available (in English). • Papers exploring evaluation of post-registration medical programs.

### Study selection

**Fig 1:**
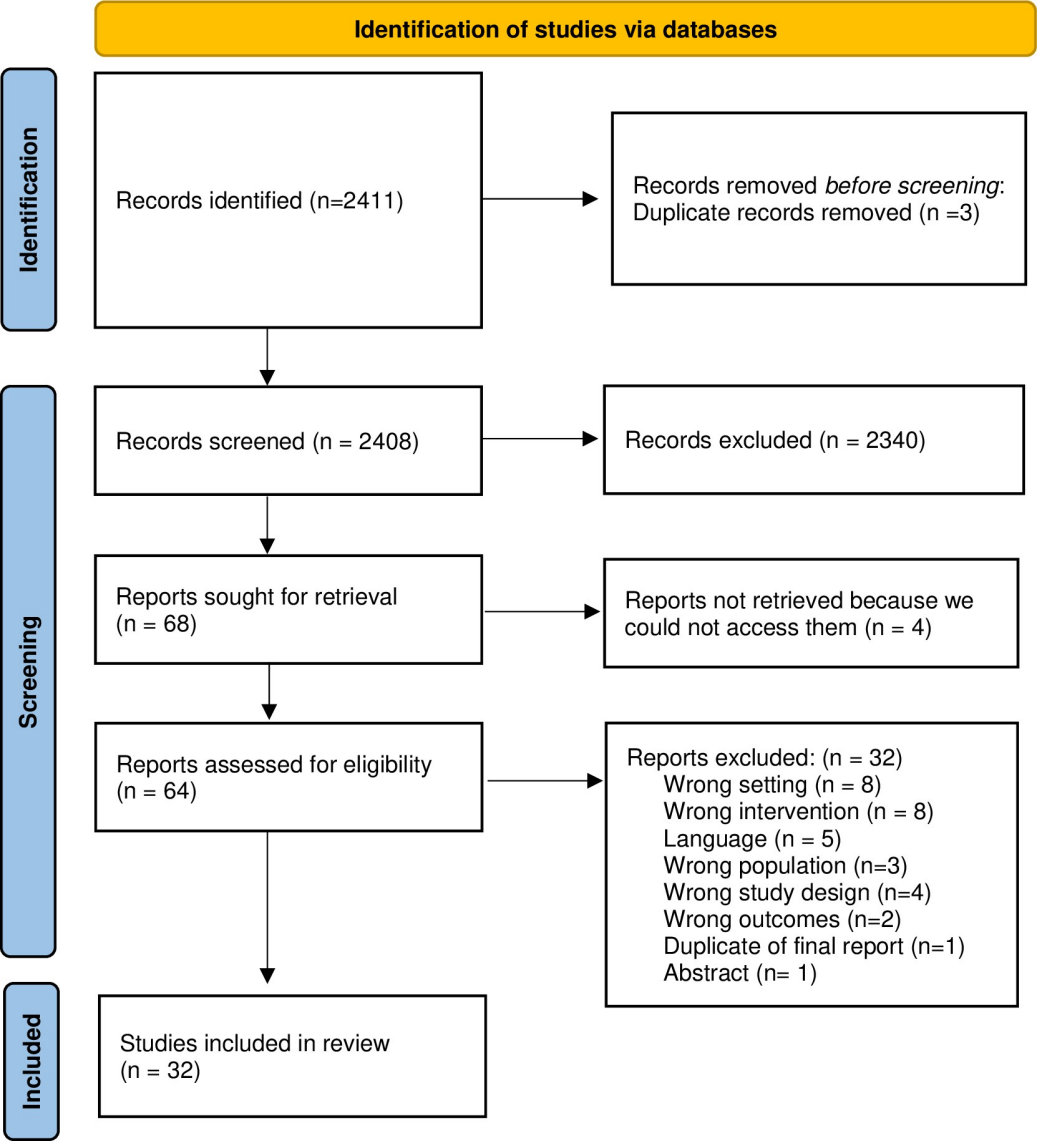
PRISMA flow diagram for study selection.

We used Covidence systematic review software to screen, collate and chart included studies (Covidence systematic review software, Veritas Health Innovation, Melbourne, Australia. Available at www.covidence.org, Australia). We completed the PRISMA-ScR (Preferred Reporting Items for Systematic Reviews and Meta-Analyses extension for Scoping Reviews) flow diagram (Fig 1) [9].

### Extraction, analysis, and screening

To extract and analyse data we followed Arksey and O’Malley’s process [7]. Title and abstract screening were conducted independently by two authors (SKG & JSP). Members of the team were assigned articles for full-text screening using Covidence Software (Melbourne VIC, Australia). Each article was screened by two members of our team (All authors). A third reviewer checked and made decisions on discrepancies (TJ). These decisions were cross-checked by two reviewers (JSP & SKG). A list of excluded studies and the reasons for exclusions are available in S2 File. Two authors (TJ & JSP) extracted data on article characteristics (including author, year, country of research, evaluation purpose, study purpose, study design and model used) and identified themes in line with the ACGME definition [1], including study purpose (apply/develop/review/validate), design, model used and whether an explicit model was referenced. In addition to this, as per Arksey and O’Malley’s guide for collating and summarizing information [7], two authors (TJ & JSP) identified qualitative patterns in the included studies. We grouped findings into themes inductively and in relation to our research questions. Disagreements were discussed with a third party (SKG) and then the wider research team. The extracted data is available in S3 File. All studies identified in the search is available in S4 File.

### Research team positionality & reflexivity

The research team comprised medical professionals and nurses, social and biomedical scientists, all of whom currently work in medical education in Australia. Some of the research team have extensive experience in curriculum design (TJ, JB, MM, JSP) and program evaluation (JB, ML). We hold a perspective that the purpose of healthcare education is both to improve the quality of healthcare and to promote the professional self-development of healthcare professionals.

From 2261 identified studies, thirty-two were included for review using the inclusion criteria outlined in Table 1. These characteristics of the included studies are shown in Table 2.

**Table 2:** Characteristics of the included studies.

Author (reference)	Year	Country	Evaluation purpose	Study purpose	Study design (methods)	Model used is stated	Model used (as defined by study authors)
Akdemir [10]	2020	Multiple	Improve	Validate a model	Utility-focused evaluation (Qualitative)	Yes	Continuous Quality Improvement
Allen [6]	2022	Multiple	Improve	Review literature	Systematic review	Yes	Kirkpatrick
Colbert-Getz [11]	2021	USA	Prove	Apply a model	Report outcomes (Quantitative)	Yes	Systems model
Fetterman [12]	2010	USA	Improve	Apply a model	Reflection (No reported methods)	Yes	Empowerment Evaluation
Findyartini [13]	2015	Indonesia	Prove	Validate a model	Validation study (Quantitative)	No	None. Focus is on student outcomes
Gibson [14]	2008	Australia	Improve	Develop a model	Reflection (No reported methods)	Yes	Multicomponent Model
Goldman [15]	2012	USA	Improve	Apply a model	Reflection (No reported methods)	No	N/A
Gulpinar [16]	2018	Turkey	Prove	Develop a model	Content Analysis (Qualitative)	No	N/A
Henry [17]	2002	USA	Prove	Develop a model	Reflection (No reported methods)	No	N/A
Karpa [18]	2012	USA	Prove	Apply a model	Reflection (No reported methods)	Yes	Content Analysis and Empowerment Evaluation
Lee [19]	2019	Multiple	Improve	Review literature	Narrative review	Yes	CIPP
MacCarrick [20]	2010	Multiple	Prove	Apply a model	Reflection (No reported methods)	Yes	WFME
Mirzazadeh [21]	2016	Iran	Improve	Apply a model	Longitudinal evaluation (Mixed methods)	Yes	CIPP
Musal [22]	2008	Turkey	Prove	Apply a model	Report outcomes (Mixed methods)	Yes	Mixed evaluation model
Oandasan [23]	2020	Canada	Improve	Review literature	Reflection (No reported methods)	Yes	Improvement-oriented evaluation
Rohwer [24]	2014	South Africa	Prove	Develop a model	Reflection (No reported methods)	No	N/A
Rooholamini [25]	2017	Iran	Prove	Apply a model	Descriptive (Mixed methods)	Yes	CIPP
Ruhe [26]	2013	Canada	Prove	Validate a model	Meta-evaluation (Mixed methods)	Yes	JCSEE
Santen [5]	2019	USA	Prove	Review literature	Reflection (No reported methods)	Yes	Kirkpatrick
Schiekirka [27]	2015	Multiple	Prove	Review literature	Narrative review	Yes	Multiple
Schmidmaier [28]	2010	Germany	Prove	Apply a model	Report outcomes (Quantitative)	No	None. Focus is on student outcomes
Sjostrom [29]	2019	Multiple	Prove	Validate a model	Systematic review (Qualitative)	Yes	WFME
Snell [30]	2000	Multiple	Prove	Review literature	Narrative review	No	N/A
Stalmeijer [31]	2022	Netherlands	Improve	Review literature	Consensus	No	N/A
Steketee [32]	2015	Australia	Prove	Develop a model	Reflection (No reported methods)	No	N/A
Sullivan [33]	2022	USA	Prove	Apply a model	Report outcomes (Mixed methods)	Yes	New world Kirkpatrick
Tackett [34]	2016	Multiple	Prove	Develop a model	Conceptual (Quantitative)	Yes	Logic
Tavakol [35]	2010	Multiple	Improve	Review literature	Reflection (No reported methods)	Yes	Multiple
Toosi [36]	2021	Iran	Improve	Review literature	Systematic review (Mixed methods)	Yes	CIPP
VanMelle [37]	2019	Canada	Prove	Develop a model	Consensus (Mixed methods)	No	N/A
Xiao [38]	2007	China	Prove	Apply a model	Report outcomes (Quantitative)	Yes	IIME standards
Yoo [39]	2020	Korea	Prove	Apply a model	Report outcomes (Quantitative)	Yes	CIPP

Abbreviations: CIPP: Context, input, process, product; WFME: World federation of medical education; N/A: Not applicable; JCSEE: Joint Committee on Standards for Educational Evaluation; IIME: Institute for International Medical Education

### Why were pre-registration medical programs evaluated?

Included studies were primarily concerned with either proving efficacy of their program (i.e., demonstrate performance relative to a standard) (n = 21) or improving quality of programs (n = 11) (Table 2). No studies were concerned with comparing programs across universities. The stated aims of the 32 studies were to review existing literature (n = 9), develop a new evaluation model (n = 7), or to apply (n = 12) or validate (n = 4) an existing model (or part thereof) (Table 2). The stated drivers for evaluation were continuous quality improvement [10, 12, 14, 23, 31, 39], benchmarking to national and international standards [5, 12, 38] accreditation [10, 18, 39] and quality assurance [29, 31].

### How do pre-registration medical programs get evaluated?

Twenty-two of the 32 studies (69%) explicitly identified an evaluation model they had used or would recommend (Table 2). The models most reported were Context-Input-Process-Product (CIPP) [19, 21, 25, 36, 39], Kirkpatrick [5, 33], World Federation Medical Education (WFME) [20, 29], and the Standard, by the Joint Committee on Standards for Educational Evaluation (JCSEE) [26]. One third of the included studies did not draw on an existing evaluation model.

Most studies were conducted via standardised student evaluation surveys [14, 17, 18, 22, 25, 30, 33, 36], whereas some studies reported that they used specific tools to evaluate the quality of curriculum content. For example, the Progress Test Medizin (PTM) [28] was used to evaluate quality within the clinical component of the curriculum. Steketee (2015) developed a curriculum mapping software to support and facilitate curriculum management [32]. As a component of curriculum evaluation, Findyartini (2015) assessed the validity of the collaborative Progress Tests conducted in three medical schools [13].

Tavakol et al (2010) highlighted the importance of economic analyses as a key component of program evaluation. It was suggested that cost-benefit analysis should be conducted on elements of the program including educator selection, technological tools in education, and the learning environment [35].

In another approach, Sullivan et al 2019 utilised multiple methods to evaluate their medical program, including pre-post surveys, focus groups and curriculum content analyses. To achieve this, the evaluation was funded as a standalone project to ensure the quality of a new medical program [33]. Even with this funding, they highlighted the difficulties of achieving a comprehensive program evaluation. Echoing the concerns of Mirzazadeh et al and Sullivan et al, Toosi et al highlight the time intensive nature of the CIPP model in addition to the omission of key stakeholders [36]. Fetterman described five tools for evaluation: culture of evidence, using a critical friend, encouraging a cycle of reflection and action, cultivating a community of learners and developing reflective practitioners [12].

Six (19%) studies utilised quantitative methodology [11, 13, 28, 34, 38, 39], three (9%) studies utilised qualitative methodology [10, 16, 29], seven (22%) used mixed methods [21, 22, 25, 26, 33, 36, 37], and eleven (34%) reported no methods because they were reflections and methods were not necessary [5, 12, 14, 15, 17, 18, 20, 23, 24, 32, 35].

### What aspects of pre-registration medical programs get evaluated?

Studies included in this review concentrated on whole of medical program [5, 6, 10, 18, 21, 22, 26, 31, 34, 35], medical school curricula [5, 13, 24, 33, 39], evidence-based medicine teaching [24], or clinical programs within a broader medical program [30, 37]. Within these broad purviews, studies evaluated various facets: student learning outcomes, graduate outcomes [5, 13, 14, 28], student attitudes [5], learning environment and facilities [5, 39], human resources [39], student mistreatment [5], program metrics such as attrition [5], core curricular content [16, 18, 33, 39], knowledge retention [28], competency-based medical education (CBME) program [23, 37], clinical teaching [30] and systematic course evaluation [15]. Outcomes that were evaluated were largely learning outcomes [33], student performance [12, 18, 21], student experience [5, 12, 14, 18, 21, 25], educator workload [14], social accountability [39] and cost of program [21, 35].

Meta-evaluation of medical programs involves the systematic assessment and analysis of the evaluation processes and findings of multiple evaluations conducted within the medical education domain. Instead of evaluating the medical program itself, meta-evaluation examines the evaluations performed on the program, focusing on their methods, quality, and outcomes. Four (13%) of the included papers concentrated on meta-evaluation, within the aspects of: continuous quality improvement [10]; assessment [13]; or whole-of-program [26, 29]. Ruhe et al. conducted a meta-evaluation by evaluating the McGill Evaluation model against JCSEE standards and highlighted the importance of achieving a balance between utility, feasibility, propriety, accuracy and evaluation accountability [26]. Akdemir et al. reviewed continuous quality improvement (CQI) by evaluating American and Canadian undergraduate accreditation systems and identified pros and cons of CQI [10].

Most studies used student evaluation surveys [14, 17, 18, 22, 25, 30, 33, 36] to evaluate quality, and one explored links between the program and patient outcomes [27]. Only five studies explored faculty experiences, perspectives, or educational quality [14, 20, 30, 36, 39]. For programs that implemented a content review [24, 33, 36], there was significant time and resource investment undertaken, which they noted as unsustainable.

Overall, current literature is heavily focused on evaluation of the curriculum and are mainly conducted via student-targeted survey-based methods, with restricted stakeholder involvement. Few studies reported patient perspective [27] or economic perspective as markers of quality [21]. There is very limited evaluation on staff wellbeing or workload [14]. Only four studies discussed burden of evaluation (economic, cognitive and administrative) [10, 13, 26, 29]. None of the studies identified in this review reported on diversity, equity and inclusion, or digital capability/citizenship (an individual’s ability to use digital systems) [40] strategies of medical programs.

## Discussion

Program evaluation is essential to evidence and continuously improve the quality of an educational course. To ensure medical graduates are well-prepared to serve within their communities, medical schools across the world need to periodically assess the standard of their educational programs. This scoping review explored the literature to explore why, how, and what medical programs considered for program evaluation.

### What are the costs of medical program evaluation?

Four evaluation models featured in pre-registration program evaluations: CIPP, Kirkpatrick, WFME and SJCSEE. This might suggest that they are the most popular ‘cleaning’ products available. But an important question is: to which extent does such popularity reflect their quality and value (for money)? CIPP is a generic evaluation model which can be adapted for use across a wide range of applications including but not limited to accreditation standards, curricular innovations, assessments, resources, finances, or student experience [21, 25, 26]. Drawing on the CIPP model, Mirzazadeh et al. additionally collected information about how much time and cost was associated with undertaking the evaluation. Findings from their cost analysis highlight sustainability as an important consideration for medical schools when using CIPP as the evaluation model [21]. Thus, considering value for money, the CIPP model (Table 2), was reported as more time intensive than other models [21, 36]. So, while that product may enable a more sparkling end-result, the cost is time.

### What are the strengths and weaknesses of different models/frameworks used in medical program evaluation?

The Kirkpatrick model for evaluation concentrates on establishing learners’ reactions, learning, behaviour, and their impacts on others. The included studies that utilised Kirkpatrick or the modified Kirkpatrick models reported largely on learner outcomes [5, 6, 11, 33]. However, two of the included studies concluded that although they used the Kirkpatrick model, they found it too linear and hierarchical, noting it did not consider a comprehensive perspective to understand why programs succeed or fail [6, 11]. In fact, in their review of the use of Kirkpatrick for program evaluation, Allen et al. state “the Kirkpatrick Model […] should not be the gold standard for program evaluation” [6]. Thus, since Kirkpatrick is used to assess outcomes of learning at four predetermined levels (noted above), its use is limited to the areas of curriculum or educational interventions [5, 33].

In contrast, WFME is a more prescriptive evaluation guide which includes components of curriculum, assessment, students, staff and other generic program components such as mission and values, quality assurance and governance. Therefore, WFME has been used for wider program evaluation and quality assurance [29]. JCSEE has been mainly used for wider program meta evaluations due to the focus on utility, feasibility, propriety, accuracy and accountability of evaluations, thus limiting its use in primary evaluations of core program areas [26]. While one might argue that WFME and JCSEE are standards, and not models, we found that the standards were used as a de facto model in the absence of any other prescribed approach to program evaluation. Perhaps this uncovers another phenomenon in program evaluation: that program evaluation occurs under the auspices of accreditation, and the documentation of such is seldom represented in published literature due to its sensitive nature.

### What should be the driver of medical program evaluations?

Our results raise additional questions for the medical education community. Is the purpose of program evaluation to identify the quality or success of a program? Is it to meet the requirements of accreditation bodies? We found evaluations were largely accreditation driven, reactive, episodic, and student focused. The implicit understanding that emerged from the included studies is that quality of a program can be assessed through students’ knowledge and reported satisfaction with their education. These are core measures that should be part of program evaluation. However, as Allen et al. point out, these do not explain contributing factors nor the complex interplay of curriculum content, environment, and the context of learning [6]. We agree. The current literature largely omits these considerations: satisfaction of other stakeholders, learner and staff wellbeing, equity and diversity. Few studies undertook meta-evaluation. We would encourage those in program evaluation to incorporate such considerations, which we outline further below.

### What is missing in the evaluation of pre-registration medical programs?

Staff and learner wellbeing is a significant contributor to, and determinant of program success, especially due to the increasing psychological distress, workload and burnout identified among higher education communities due to ineffective academic environments exacerbated by lack of appropriate policies [41, 42]. Markers of quality within this domain might include equitable access to support and significance given to staff/learner wellbeing within the program [43, 44]. The learning environment and culture impacts on how learners engage and perform [45]. Existing models can be utilised to consider program, faculty and universities key performance indicators (KPIs) [43].

Diversity, Equity and Inclusion (DEI) are important as markers of quality, since a diverse and equitable learner/staff body helps to model to students the needs of the heterogenous healthcare industry [46]. The included studies have not focused on inclusion or empowerment in their evaluations. The evaluation literature offers a limited picture of how medical programs are promoting DEI. Another reason for a scarcity of DEI data in evaluation literature may be the hesitancy of institutions to publish data that could be interpreted as negative or insufficient. We call for program evaluators to include evaluation of DEI strategies. For evaluators who want to include such aspects, we recommend they underpin evaluations with realism and transparency [47, 48].

None of the included studies evaluated their programs in terms of student digital capability and citizenship [40]. In the context of the recent boom in Artificial Intelligence, and accrediting bodies requiring digital capability, we also call for medical programs to evaluate strategies they have in place for promoting digital citizenship.

Meta-evaluation is important to answer the question: is the evaluation process working? We recommend medical programs undertake periodical meta-evaluation of the evaluation model, evaluation process and evaluation burden (cost, cognitive and administrative). As Tavakol et al. suggest, evaluations should ensure there is a return on investment for the medical program delivered [35]. Meta-evaluation was the focus of four articles [10, 13, 26, 29], in which financial, cognitive and administrative burden of evaluation has not been covered. In contrast, when studies concentrate on meta-evaluation of the financial burden [21, 35], aspects of evaluation process, cognitive and administrative burden are largely omitted. It is vital to compromise between conducting effective evaluation and evaluation burden, in a resource limited health/ higher education system, where time spent on evaluation could compete with time spent in teaching and clinical care.

### Who should be involved in the evaluation of pre-registration medical programs and when?

In terms of stakeholders, we suggest that qualified practicing healthcare professionals and consumers of healthcare are also key stakeholders who can offer important insights into the quality of a given medical program. Quality improvement science suggests that there is a continuum of stakeholder engagement: non-participating, symbolic, and engaged participation [49]. This continuum proposes that there are varying levels of stakeholder engagement, that range from information sharing to collaborative patient engagement models, such as community-based participatory research. Stakeholders with different levels of engagement offer different perspectives on the quality of the program. Medical programs might source broader stakeholder engagement from university academics or professional staff (internal and external to the program e.g. accrediting bodies), adjunct clinical professors, hospital-based clinicians involved in education delivery, simulated participant programs and healthcare consumers (patients and their carers).

### How should we implement program evaluation?

In terms of the logistics of program evaluation, authors who used the CIPP model highlighted the time-intensive nature of comprehensive program evaluation. This implores the question of how often program evaluation should occur and what should trigger a more comprehensive review? None of the included papers described a timing cycle for review, nor did they identify thresholds to trigger ad hoc reviews. We propose that accreditation should be the key driver for timing of reviews and should be considered the “proactive” model of program evaluation, as many require annual reporting. If this is not practicable, a 3-yearly cycle may be more appropriate to accommodate for changing science or practice relevant to pre-registration medicine programs. As for reactive models, educators might consider key issues such as: 1) consistent, significant worsening student performance outcomes, 2) consistent, widespread student dissatisfaction over a period, 3) resourcing constraints (including physical environments, educational tools and staffing), and/or 4) significant shifts in evidence or knowledge of fields of science.

The current focus of pre-registration medical program evaluation literature is limited to one or two facets of a program. Evaluations are often accreditation-driven, giving utmost prominence to student satisfaction at the expense of other stakeholders. The main areas frequently evaluated are curriculum, learning outcomes and graduate outcomes, learning environments, program metrics, student performance and student experience. Evaluation of staff, well-being, DEI, digital citizenship, finances, and meta evaluation are quite overlooked (dusty) areas within the literature and need attention. Use of broad stakeholder involvement as highlighted above, is encouraged for any evaluation.

### Strengths and limitations

We searched a wide range of literature sources on evaluation published since 2000. This allowed us to look at a range of medical education literature and retrieve articles from various countries. However, this also meant that we did not include unpublished evaluation research, which is likely where significant amounts of program evaluation data are reported. We found designing a search strategy for program evaluation quite difficult, due to the variety of methods and terminology used for program evaluation. This challenge meant crafting a search strategy that was focused on the inclusion of the terms “program” and “evaluation”, and thus, our search may not have identified papers that used different terms. Additionally, we restricted our search to English language journal articles and thus may have excluded relevant research in languages other than English.

## Conclusion

Program evaluation is more than mere housework. It is fundamental to driving the quality of education delivered for workforce ready healthcare professionals. Current evaluations tend to focus on student experience and content delivery. There are significant gaps in existing literature on evaluation related to staff, learner/staff well-being, equity, diversity, and meta evaluation. We recommend those involved in program evaluation to consider these areas when planning program evaluations.

## Supporting information

S1 File Search string. (DOCX)

S2 File Studies excluded at the full text screening stage and reasons for exclusion. (DOCX)

S3 File Extracted data. (CSV)

S4 File All studies identified in the literature search. (CSV)
