# Supplementary material for: Internal evaluation of medical programs is more than housework: A scoping review
Source: PLoS One. 2024 Oct 25;19(10):e0305996. doi: 10.1371/journal.pone.0305996 (PMC11508059; doi:10.1371/journal.pone.0305996)
Supplement: S1 File — (DOCX) [file pone.0305996.s001.docx]

Supplementary Material

Search Strategy:

(((exp "Program Evaluation"/ OR Evaluation.tw.)

AND

(Strategies OR Standards OR Approach OR Approaches OR Framework OR Frameworks OR Process OR Processes OR Categorize OR Categorise OR Checklists OR Modification).tw.

AND

(exp "Education, Medical"/ OR "Medical Education".tw. OR "Medical School".tw. OR "Health Professions Education".tw. OR "Public health".tw.)

AND

(exp Curriculum/ OR Curriculum.tw. OR Curricula.tw. OR Curricular.tw. OR Programme.tw. OR Programmes.tw. OR Programs.tw.)

AND

((Outcomes OR Outcome OR Course OR Competence OR Competency OR Practical OR Quality) (Measured OR Measure OR Measuring OR Increases OR Improved OR Improvement OR Improvements OR Impact OR Better OR Factors OR Desired OR Implementation OR Cost OR Metrics)).tw.) OR (((Evaluation OR Standards) and (Medical or Clinical) and (Education or Curriculum)).ti.)

AND

(exp "Competency-Based Education"/ OR exp "Outcome Assessment, Health Care"/ OR "Expert Panel".tw. OR Stakeholders.tw. OR Committee.tw. OR Education.ti. OR Groups.tw. OR Guide.tw. OR Investigated.tw. OR Analysis.tw. OR Statistics.tw. OR Data.tw. OR Validity.tw. OR Cost.tw.))
