# Supplementary material for: Internal evaluation of medical programs is more than housework: A scoping review
Source: PLoS One. 2024 Oct 25;19(10):e0305996. doi: 10.1371/journal.pone.0305996 (PMC11508059; doi:10.1371/journal.pone.0305996)
Supplement: S2 File — (DOCX) [file pone.0305996.s002.docx]

| Title | Authors | Published Year | Journal | Covidence # | Study | Notes | Tags | |
| --- | --- | --- | --- | --- | --- | --- | --- | --- |
| From quality assurance to total quality management: how can quality assurance result in continuous improvement in health professions education? | Dolmans, D. H.; Wolfhagen, H. A.; Scherpbier, A. J. | 2003 | Education for Health | #78 | Dolmans 2003 | Exclusion reason: Full text unavailable; |  |  |
| Scientific evaluation - The basis for quality assurance in continuing medical education | Haffner, C.; Giere, W.; Loch, E. G.; Rieck, G. | 2006 | Zeitschrift fur Arztliche Fortbildung und Qualitatssicherung | #109 | Haffner 2006 | Exclusion reason: Wrong setting; |  |  |
| Outcomes of competency-based medical education: A taxonomy for shared language | Hall, A. K.; Schumacher, D. J.; Thoma, B.; Caretta-Weyer, H.; Kinnear, B.; Gruppen, L.; Cooke, L. J.; Frank, J. R.; Van Melle, E. | 2021 | Medical teacher | Hall 2021 | Exclusion reason: Wrong setting; |  |  |  |
| An institutional system to monitor and improve the quality of residency education | Heard, J. K.; O'Sullivan, P.; Smith, C. E.; Harper, R. A.; Schexnayder, S. M. | 2004 | Academic Medicine | Heard 2004 | Exclusion reason: Wrong setting; Joan Roehl (2022-08-24 13:56:29)(Select): graduate medical education (residency); |  |  |  |
| The role of program evaluation in medical education at the John A. Burns School of Medicine | Kasuya, R.; Arakaki, L.; Linberg, M.; Sakai, D. | 2003 | Hawaii medical journal | #143 | Kasuya 2003 | Exclusion reason: Full text unavailable; |  |  |
| Exploring the connections between programmatic assessment and program evaluation within competency-based medical education programs | Moreau, K. A. | 2021 | Medical teacher | Moreau 2021 | Exclusion reason: Wrong intervention; |  |  |  |
| Medical education quality evaluation | Niksic, D.; Cemerlic, A.; Bajraktarevic, S. | 2001 | Medicinski arhiv | #203 | Niksic 2001 | Exclusion reason: Full text unavailable; |  |  |
| Evaluation of the medical curriculum: Why, when, by whom and for whom should questionnaires be used | Pabst, R.; Nave, H.; RothkÃ¶tter, H. J.; Tschernig, T. | 2001 | European Journal of Morphology | Pabst 2001 | Exclusion reason: Full text unavailable; Joan Roehl (2022-08-24 13:47:51)(Select): The uploaded file is not the full text.; |  |  |  |
| A guide for evaluation of online learning in medical education: a qualitative reflective analysis | Wasfy, N. F.; Abouzeid, E.; Nasser, A. A.; Ahmed, S. A.; Youssry, I.; Hegazy, N. N.; Shehata, M. H. K.; Kamal, D.; Atwa, H. | 2021 | BMC medical education | Wasfy 2021 | Exclusion reason: Wrong setting; Joan Roehl (2022-08-24 13:31:51)(Select): emphasis on online learning; |  |  |  |
| STUDENTJAMA. The evolution and evaluation of modern medical education | Staropoli, J. F. |  | 291 | Staropoli 2004 | Exclusion reason: Wrong setting; Joan Roehl (2022-08-24 14:17:23)(Select): This is just a one-page editor's note; |  |  |  |
| Towards effective evaluation and reform in medical education: a cognitive and learning sciences perspective | Patel, V. L.; Yoskowitz, N. A.; Arocha, J. F. | 2009 | Advances in Health Sciences Education | Patel 2009 | Exclusion reason: Wrong intervention; |  |  |  |
| How good are we? An evaluation of the quality of medical education | Bengtsson, A.; Johansson, K. |  | 98 | Exclusion reason: Wrong setting; |  |  |  |  |
| Global standards and accreditation in medical education: A view from the WFME | Karle, H. | 2006 | Academic Medicine | Karle 2006 | Exclusion reason: Wrong study design; |  |  |  |
| It's time to define the Swedish Doctor? Lessons learnt from the evaluation of the Finnish undergraduate medical education | MÃ¶ller, R. |  | 116 | MÃ¶ller 2019 | Exclusion reason: Full text unavailable; |  |  |  |
| Building the evidence base: Networking innovative socially accountable medical education programs | PÃ¡lsdÃ³ttir, B.; Neusy, A. J.; Reed, G. | 2008 | Education for Health: Change in Learning and Practice | #405 | PÃ¡lsdÃ³ttir 2008 | Exclusion reason: Wrong outcomes; |  |  |
| National survey of evaluation practices and performance-guided resource allocation at German medical schools | Schiekirka-Schwake, S.; Barth, J.; Pfeilschifter, J.; Hickel, R.; Raupach, T.; Herrmann-Lingen, C. | 2019 | German medical science : GMS e-journal | Schiekirka-Schwake 2019 | Exclusion reason: Wrong outcomes; |  |  |  |
| WFME task force on defining international standards in basic medical education. Report of the working party, Copenhagen, 14-16 October 1999 | Anonymous, | Aug | 34 | Anonymous 2000 | Exclusion reason: Preliminary report -duplicate of final report; |  |  |  |
| A practical guide to using the World Federation for Medical Education standards. WFME 2: Educational program | MacCarrick, G. | 2010 | Irish Journal of Medical Science | MacCarrick 2010 | Exclusion reason: Wrong outcomes; |  |  |  |
| Aggregated student confidence estimates support continuous quality improvements in a competencies-oriented curriculum | Papa, F. J.; Alexander, J. H. | 2019 | BMJ open quality | Papa 2019 | Exclusion reason: Wrong intervention; |  |  |  |
| Standards in medical education - Keeping up with whom? | van de V Niekerk, J. P. |  | 90 | vandeVNiekerk 2000 | Exclusion reason: Full text unavailable; |  |  |  |
| AM last page: A snapshot of three common program evaluation approaches for medical education | Blanchard, R. D.; Torbeck, L.; Blondeau, W. |  | 88 | Blanchard 2013 | Exclusion reason: Wrong setting; Joan Roehl (2022-08-24 14:05:34)(Select): poster; |  |  |  |
| An anticipatory quality improvement process for curricular reform | Hollander, H.; Loeser, H.; Irby, D. | 2002 | Academic medicine : journal of the Association of American Medical Colleges | #660 | Hollander 2002 | Exclusion reason: Wrong intervention; |  |  |
| Evaluating the performance of medical educators: A novel analysis tool to demonstrate the quality and impact of educational activities | Chandran, L.; Gusic, M.; Baldwin, C.; Turner, T.; Zenni, E.; Lane, J. L.; Balmer, D.; Bar-On, M.; Rauch, D. A.; Indyk, D.; Gruppen, L. D. | 2009 | Academic Medicine | Chandran 2009 | Exclusion reason: Wrong setting; |  |  |  |
| A conceptual model for program evaluation in graduate medical education | Musick, D. W. | 2006 | Academic Medicine | Musick 2006 | Exclusion reason: Wrong setting; |  |  |  |
| Evaluation of teaching and learning: A basis for improvement in medical education | Yang, X. F.; Talmy, T.; Zhu, C. H.; Li, P. F.; Wang, W.; Zhang, P.; Zhang, H. W.; Bulis, S.; Wang, K. X.; Chen, X.; Wang, Y. L.; Jiang, D. P.; Zong, Z. W.; Zhou, J. |  | 130 |  |  |  |  |  |
| Evaluation in medical education | Yoon, T. Y. |  | 27 | Exclusion reason: Full text unavailable; Lucy Grant (2022-08-23 14:05:29)(Select): need english language version?; |  |  |  |  |
| Quality of undergraduate, graduate and continuing medical education | Adler, G.; Von Dem Knesebeck, J.; HÃ¤nle, M. M. | 2008 | Zeitschrift fur Evidenz, Fortbildung und Qualitat im Gesundheitswesen | Adler 2008 | Exclusion reason: Language; Joan Roehl (2022-08-24 13:49:35)(Select): Full text is in German. Can help to translate if needed.; |  |  |  |
| Evaluation of existing curriculum (2002) of undergraduate medical education in Bangladesh | Hussain, M. M.; Talukder, H. K.; Moazzem, N.; Rashid, A.; Hanif, A.; Nargis, T. | 2011 | Mymensingh medical journal : MMJ | #934 | Hussain 2011 | Exclusion reason: Wrong study design; |  |  |
| Implementing a Course Review Process for a Continuous Quality Improvement Model for a Medical School Curriculum | Ward, C. S.; Andrade, A.; Walker-Winfree, L. | 2018 | Journal of health care for the poor and underserved | Ward 2018 | Exclusion reason: Full text unavailable; |  |  |  |
| Social Accountability Frameworks and Their Implications for Medical Education and Program Evaluation: A Narrative Review | Barber, C.; van der Vleuten, C.; Leppink, J.; Chahine, S. | 2020 | Academic medicine : journal of the Association of American Medical Colleges | Exclusion reason: Wrong setting; |  |  |  |  |
| Fostering Student-Faculty Partnerships for Continuous Curricular Improvement in Undergraduate Medical Education | Scott, K. W.; Callahan, D. G.; Chen, J. J.; Lynn, M. H.; Cote, D. J.; Morenz, A.; Fisher, J.; Antoine, V. L.; Lemoine, E. R.; Bakshi, S. K.; Stuart, J.; Hundert, E. M.; Chang, B. S.; Gooding, H. | 2019 | Academic medicine : journal of the Association of American Medical Colleges | Scott 2019 | Exclusion reason: Wrong outcomes; |  |  |  |
| Using Contribution Analysis to Evaluate Competency-Based Medical Education Programs: It's All About Rigor in Thinking | Van Melle, E.; Gruppen, L.; Holmboe, E. S.; Flynn, L.; Oandasan, I.; Frank, J. R. | 2017 | Academic medicine : journal of the Association of American Medical Colleges | VanMelle 2017 | Exclusion reason: Wrong intervention; |  |  |  |
| Analytical case study of evaluation of curriculum at a medical school | An, J. H.; Han, J. J.; Kim, N. J.; Eo, E.; Kwon, I.; Lee, S. N. | 2010 | Korean Journal of Medical Education | An 2010 | Exclusion reason: Language; Joan Roehl (2022-08-24 15:15:01)(Select): English version?; |  |  |  |
| Principles of medical education, educational objectives and evaluation strategy | AtaoÄŸlu, S. |  | 20 | Exclusion reason: Language; |  |  |  |  |
| The frankfurt concept of evaluation of teaching in medical education | HÃ¶velmann, R.; Kersken-NÃ¼lens, U.; LohÃ¶lter, R.; Drolshagen, S.; Korf, H. W. | 2001 | Medizinische Ausbildung | #1541 | HÃ¶velmann 2001 | Exclusion reason: Full text unavailable; |  |  |
| Research and development of evaluation criteria for premedical curriculum | Hwang, J.; Shin, J. S.; Yoon, H. B.; Kim, D. H.; Yoo, D. M.; Kim, E. J.; Lee, S. H. | 2015 | Korean journal of medical education | Hwang 2015 | Exclusion reason: Language; Joan Roehl (2022-08-24 13:16:09)(Select): English version?; |  |  |  |
| Evaluation of Competency Based Medical Education Curriculum | Ilhan, Elif | 2021 |  | #1557 | Ilhan 2021 | Exclusion reason: Full text unavailable; |  |  |
| Medical education - beyond the evaluation of intellectual competency | Kim, S. | 2011 | Korean Journal of Medical Education | Kim 2011 | Exclusion reason: Language; Joan Roehl (2022-08-24 15:06:17)(Select): English version?; |  |  |  |
| Evaluating a Continuing Medical Education Program: New World Kirkpatrick Model Approach | Liao, Shih-Chieh; Hsu, Shih-Yun | Journal of Management, Economics and Social Sciences 2019 | 4 | Exclusion reason: Wrong setting; Joan Roehl (2022-08-24 13:19:55)(Select): "The acupuncture training program is specially designed for physicians and dentists..."; |  |  |  |  |
| Accreditation System and Standards for Medical Education in Pakistan: It's time we raise the bar | Sethi, A.; Javaid, A. | Nov-Dec | 33 |  |  |  |  |  |
| Evaluation of medical education programs: Need, scope, and tools | Shrivastava, S.; Shrivastava, P. |  | 18 | Exclusion reason: Wrong intervention; |  |  |  |  |
| Commitment to Change Instrument Enhances Program Planning, Implementation, and Evaluation | White, Marc I.; Grzybowski, Stefan; Broudo, Marc | 2004 | 24 |  | Exclusion reason: Wrong outcomes; |  |  |  |
| Dental education evaluation in China: a systematic review | Yang, J.; Zhang, Y.; Ye, X.; Xie, C.; Ge, X.; Lu, F.; Yu, Q.; Sun, H. | 2014 | BMC medical education | Yang 2014 | Exclusion reason: Wrong setting; Joan Roehl (2022-08-24 14:12:00)(Select): dental education; |  |  |  |
|  |  |  |  |  |  |  |  |  |
| Evaluation of a competency based medical curriculum in a Sub-Saharan African medical school | McKenzie-White, J.; Mubuuke, A. G.; Westergaard, S.; Munabi, I. G.; Bollinger, R. C.; Opoka, R.; Mbalinda, S. N.; Katete, D.; Manabe, Y. C.; Kiguli, S. | 2022 | BMC medical education | #17 | McKenzie-White 2022 | Exclusion reason: Educational intervention only; |  |  |
| Quality Assurance of Undergraduate Medical Education in Israel by Continuous Monitoring and Prioritization of the Accreditation Standards | Benbassat, Jochanan; Baumal, Reuben; Cohen, Robert | 2022 | Rambam Maimonides medical journal | #35 | Benbassat 2022 | Exclusion reason: Wrong study design; |  |  |
| Evaluation of the doctoral program in medical education of Isfahan University of Medical Sciences based on the CIPP model | Derakhshanfard, Samira; Jokar, Fariba; Ehsanpour, Soheyla; Hadadgar, Arash | 2022 | Journal of education and health promotion | #53 | Derakhshanfard 2022 | Exclusion reason: Wrong population group; |  |  |
| Student evaluation of medical school curriculum transformation in Iraq | Jawad, Huda Noori; Abd-Alnabi, Zainab Amir; Abd-alKadir, Layla Mohammed; Hassan, Noor Falah; Mutlaq, Zahraa Abbas; Doshi, Krishna; Kron, Michael; Alhaidari, Taghreed K. | 2020 | MedEdPublish (2016) | #79 | Jawad 2020 | Exclusion reason: Wrong study design; |  |  |
| Health professions education in Serbia: evaluation and measures for quality improvement through experiential education, interprofessional education, and teaching competencies development | KovaÄeviÄ‡, M.; OdaloviÄ‡, M.; DjukiÄ‡-Ä†osiÄ‡, D.; VasiljeviÄ‡, D.; ParojÄiÄ‡, J.; TasiÄ‡, L. | 2022 | Vojnosanitetski Pregled | #86 | KovaÄeviÄ‡ 2022 | Exclusion reason: Wrong population group; |  |  |
| Scenario-Based Learning: Preliminary Evaluation of the Method in Terms of Students' Academic Achievement, In-Class Engagement, and Learner/Teacher Satisfaction | Mamakli, Sumer; Alimoglu, Mustafa Kemal; Daloglu, Mustafa | #95 |  | Exclusion reason: Educational intervention only; |  |  |  |  |
| Using Clinical Narratives in Program and Curriculum Evaluation | Melillo, C.; Chavez, M.; Powell-Cope, G.; Rugs, D.; Cowan, L.; Shaw, S. M.; Barrett, B.; Rugen, K. W. | 2020 | Journal of health science & education | #99 | Melillo 2020 | Exclusion reason: Wrong population group; |  |  |
| Appreciative Inquiry Approach in Evaluation of Medical Education Programs | Salajegheh, M. | 2022 | Journal of Isfahan Medical School | #124 | Salajegheh 2022 | Exclusion reason: Wrong study design; |  |  |
| EVALUATION OF CLINICAL SKILLS ASSESSMENT PROGRAM AT NYU GROSSMAN SCHOOL OF MEDICINE: OPPORTUNITIES FOR FUTURE DIRECTIONS IN CURRICULUM REDESIGN | Schaye, V.; Kudlowitz, D.; Robak, M.; Crowe, R.; Zabar, S. R.; Gillespie, C.; Harnik, V.; Rosenfeld, M.; Crotty, K. | 2022 | Journal of General Internal Medicine | #125 | Schaye 2022 | Exclusion reason: Abstract; |  |  |
